# Combination of Passive and Active Solar Heating with Thermal Energy Storage

**DOI:** 10.3390/molecules27144386

**Published:** 2022-07-08

**Authors:** Andreas Thangam, Amar Auckaili, Mohammed Farid

**Affiliations:** Chemical and Materials Engineering, The University of Auckland, Auckland 1142, New Zealand; akhu002@aucklanduni.ac.nz (A.T.); a.auckaili@auckland.ac.nz (A.A.)

**Keywords:** thermal energy storage, phase change materials

## Abstract

This study investigated the impact of individual and combination of different sources of heating (passive solar heating, electric oil-heater, and solar air heater) in a pilot-scale building containing phase change material (PCM) for a potential reduction in energy consumption while maintaining thermal comfort. Unlike most of the recent simulations and modelling studies, this impact was tested experimentally using two identical control and test huts located at the University of Auckland. The control hut was equipped with standard gypsum boards while the test hut had gypsum boards containing PCM (PureTemp 20, PT20). The study found that combining both active and passive solar heating with a temperature-controlled electric oil heater demonstrated the ability to provide significant energy savings and maintain thermal comfort in the test hut, most notably overnight. The suggested combination was tested over different weather conditions and with different temperature constraints to maintain thermal comfort and achieve energy savings ranging from 33% to 87.5%.

## 1. Introduction

As a common scholarly finding among many studies, it is imperative to bridge the gap between the non-environmentally friendly, limited energy production and excessive consumption by investing in the applications of thermal energy storage [1,2]. Technical analysis of the global energy consumption highlighted the excessive consumption of energy in the building sector [3], of which significant improvements could be made to the HVAC operations [4]. Through the process of conducting many research projects, as published in “*Thermal Energy Storage with Phase Change Materials*” [5], the authors examined the factors affecting the buildings of lightweight materials and diagnosed the important role played by the thermal mass of the building [6,7] and the lack of thermal mass present in lightweight building materials [8]. The authors of the same book [5] also answered the core question of how the thermal mass of new and existing buildings, particularly those made from lightweight materials, can be increased. This question was answered through the experimental examination and modelling of the mechanisms by which thermal energy is stored and notably latent heat storage using phase change materials (PCM) [9,10]. The incorporation of PCM in buildings is an application that has constantly been investigated over the last 30 years, yet there are no wide commercial applications for it [11]. The ways in which the PCMs integrated into buildings including immersion, microencapsulation, and macro encapsulation [12,13,14] had been researched in a wide range of applications including PCM in walls [15], underfloor heating systems [16] and space cooling systems [17].

Most of the scholarly findings came to the same advantages of using PCM as a means of improving the thermal mass of lightweight buildings despite including a range of methods (experimental, modelling and simulation) and locations [18]. Due to this convergence in the findings, the following impacts of increasing thermal mass can be reported:

- Reduction in indoor temperature fluctuations and maintaining thermal comfort [5,7,9];
- Reduction in energy consumption for heating/cooling and efficient use of HVAC units [2,17];
- Enabling peak-load shifting of electricity and producing significant energy savings [19,20]. Combined with lower prices being available during off-peak periods, the associated cost savings could provide the public with an incentive to use PCM and improve the thermal performance of their buildings [19,20];
- Improve the feasibility and distribution of both passive and active solar heating. The intermittent nature of solar heating could be compensated by the thermal energy storage of the PCM present in the building [21].

Despite the large experimental work conducted on PCM applications with the most potential in buildings, there is little work published on using a combination of passive and active solar heating in an in-situ experimental facility. This paper focused on studying this mechanism with an electric source of heating in a lightweight building structure containing PCM. Subsequently, the key objective of this study is to achieve energy savings and maintain thermal comfort using solar energy and PCM during winter in the city of Auckland, New Zealand. This objective includes exploring the capabilities of solar heating in different ways and assessing the effectiveness of the suggested mechanisms.

## 2. Experimental Set-Up

**Figure 1:**
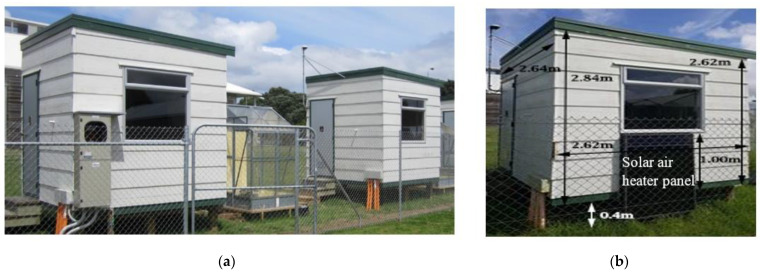
(**a**) The experimental test and control huts, located at the University of Auckland [22]. (**b**) The hut was supplied with a solar air heater panel [19].

The experiments detailed in this paper took place in two identical test and control huts located at the University of Auckland, and a third hut was used for data collection. The huts were made of lightweight building materials with internal dimensions of 2.4 × 2.4 × 2.4 m, as published by Amar Auckaili in his Ph.D. Thesis submission [22] and shown in Figure 1.

Hut-1 was considered the ‘control hut’ and had standard 13 mm thick gypsum boards lining the interior walls and ceiling. Hut-2 was considered the ‘test hut’ where the walls and ceilings were the same as Hut-1 but contained 22-wt% of the PCM (PT20) from PureTemp. The macro-encapsulation of the PCM within boards had been conducted using a direct immersion technique. PT20 is 100% renewable and has a heat storage capacity of ∆H = 180 J/g and a peak melting temperature of 20 °C. The solar air heaters with north-facing photovoltaic panels had been installed outside the huts. Different configurations of an individual and a combination of heating supplies were explored in this project. While the passive solar heating was provided by solar irradiation, the solar air heaters were used in certain configurations to provide a form of active solar heating. Both huts were also equipped with 850 W electrically powered oil heaters, which followed a control method of temperature constraints. A complete set of indoor and outdoor thermocouples were located throughout and outside the huts with special care being taken not to be in contact with the wall or direct exposure to sunlight. All temperature data, along with the energy consumption of the heaters (as monitored by a Cerrel Electrade LP-1W1), were subsequently recorded every minute using LabView software. The period of data recording was continued over two months between 30 June 2018 and 1 September 2018.

## 3. Results and Discussion

### 3.1. Performance of PCM with Passive Solar Heating

**Figure 2:**
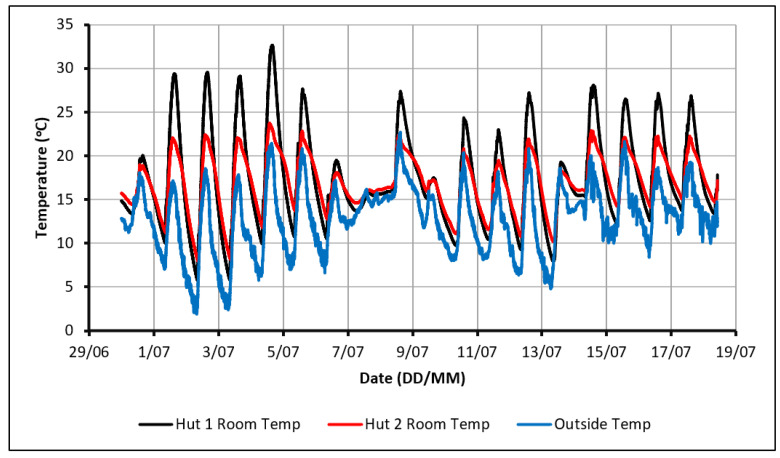
Temperature profiles in case of using passive solar heating only.

The initial experiment consisted of monitoring the temperature profiles within the huts over a range of weather conditions without supplying any external heat other than solar radiation. Although it was not expected that the PCM could make a significant impact on the comfort levels within Hut-2 without the use of some active heating, this experiment would provide a greater understanding of the behavior of the PCM in the experimental set-up, highlighting points and trends of interest for future experiments. The indoor and outdoor temperature profiles over 18 days can be seen in Figure 2 with the maximum and minimum outdoor temperatures experienced being 22.5 °C and 2 °C, respectively.

The temperature profiles show that the indoor temperature in Hut-1 reached a higher peak than that observed in Hut-2. This difference in peak temperatures indicates that the PCM was successful in absorbing heat during the daytime. In most of the readings, Hut-1 exceeded 27 °C, which is considered an uncomfortable dwelling temperature while Hut-2 maintained a relatively comfortable peak around 23 °C. When cooling down at night-time, the indoor temperature in Hut-1 dropped at a faster rate than that observed in Hut-2. This was due to the discharge of thermal energy from the PCM, which had been stored during the day. Consequently, Hut-2 remained warmer than Hut-1 in the night-time, although the temperatures were still below comfortable limits. It can also be seen in Figure 2 that, on several days, the PCM did not rapidly charge and the temperatures in both huts would have been nearly identical due to the cloudy weather (poor heating conditions). This impact of PCM existence in reducing the fluctuation of indoor temperature confirms that the PCM was working as intended if sufficient passive heating was available. Despite this limited positive impact, it is believed that a source of active heating is required if comfortable conditions are to be achieved constantly. This heat source would need to supplement heating during the day to fully charge the PCM, keep the huts warm on days of poor outdoor conditions and prevent excessively low temperatures at night.

### 3.2. Performance of PCM with Passive Heating and Indoor Electric Heating

To address the need for supplementary heating identified in the previous section, the effectiveness of two potential heat sources in conjunction with the PCM and passive solar heating was tested in both huts. The first was the 850 W oil heater and the second was the solar air heater. The aim of this was to determine how well each worked with the PCM in terms of maintaining thermal comfort and using energy efficiently.

#### 3.2.1. Use of Temperature-Controlled Electric Oil Heater

**Figure 3:**
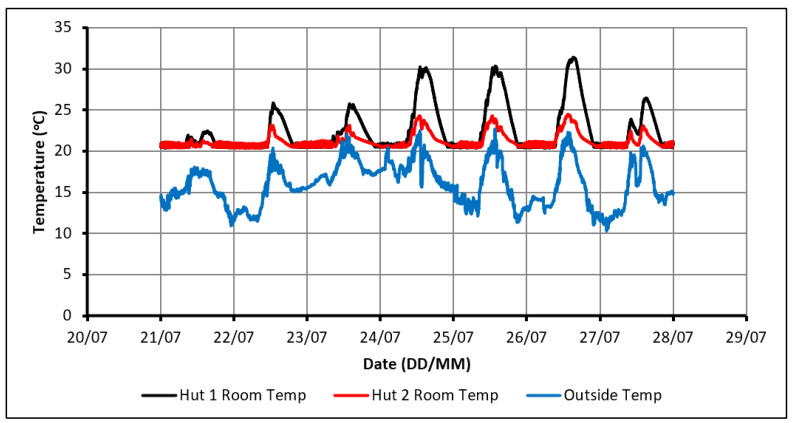
Temperature profiles in case of using passive solar heating and oil heater.

The control strategy of temperature was set up so that the heater would maintain the indoor temperature within the range of 20 °C to 22 °C. This range was chosen to be within the comfortable limits and would partially charge the PCM overnight to ensure complete charging even on cloudy days of poor sunlight. Figure 3 shows that the room temperature in the huts did not drop below 20 °C. This confirms that the control system was operating as intended. It can also be observed that Hut-1 experienced a higher peak indoor temperature than Hut-2 every day. This can once again be attributed to PCM absorbing the heat, preventing the indoor temperature of Hut-2 from rising above 24.5 °C. On the other hand, the temperature in Hut-1 has risen to 30 °C on several days. Therefore, when solely considering thermal comfort, this configuration could be suitable for consideration.

**Figure 4:**
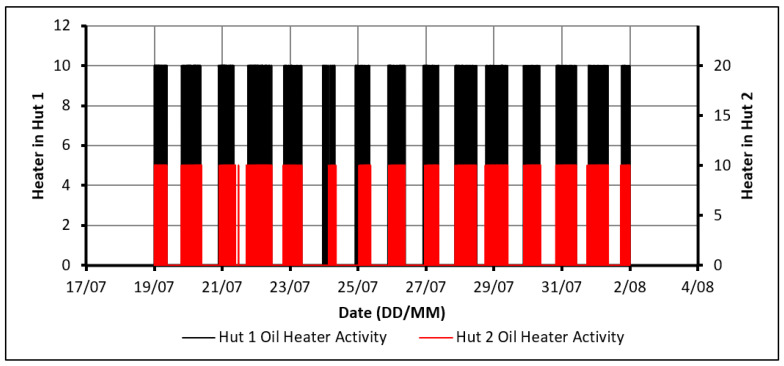
Activity of the oil heaters.

The activity of the heaters in both huts is displayed in Figure 4. An activity value of 10 shows that the heater was on, while 0 means it was off. From the detailed hourly data of this figure, it was determined that all the heating in both huts took place between the hours of 5:00 p.m. and 11:30 a.m. the next day.

The drawback of using this configuration arises when examining the power consumption between the huts. Over the 14 days, Hut-2 consumed 1% more energy than Hut-1. Although this value could be deemed negligible, it would still indicate that the PCM provides no noticeable improvement in this regard. It, therefore, fails to meet one key project objective, which is to save energy using PCM. It is believed that the main cause of this failure is the temperature constraint chosen. While the PCM begins to discharge and decreases the rate of cooling in Hut-2, both huts hit the user-specified temperature constraint of approximately 21 °C at a very similar time. Therefore, both huts used the heater over approximately the same time. However, as the PCM was being charged over this period, Hut-2 consumed more energy than Hut-1. This highlights the need to give the PCM sufficient time to discharge to make noticeable energy savings.

#### 3.2.2. Use of Solar Air Heater

**Figure 5:**
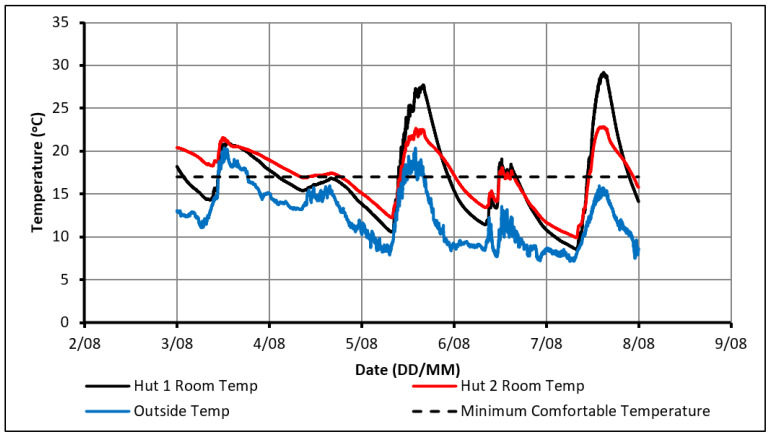
Temperature profiles in case of using passive solar heating and solar air heater.

For the second part of this section, the electric oil heaters were turned off, and the solar air heaters were activated. Figure 5 shows that, on 3 of 5 days, the use of the solar air heater managed to elevate the indoor temperature of the huts above 20 °C. Overheating was observed in Hut-1 on days 3 and 5 but was avoided in Hut-2 because the heat was absorbed by the PCM. In this respect, it can be argued that the use of a solar air heater is not viable without the presence of the PCM.

The indoor temperature of Hut-2 never dropped below 17 °C between 11:30 a.m. and 3:30 p.m., suggesting that minimal daytime heating would be required from a supplementary source. The benefit of having the PCM is further emphasized as Hut-2 did not hit the same daily low temperatures as Hut-1, which was a result of a noticeably slower rate of cooling. Both observations can once again be attributed to the discharge of the stored energy from the PCM overnight. All of this not only indicates that the solar air heaters have the potential to provide a comfortable level of warmth during the day when used with the PCM, but that they can also provide sufficient charge to delay and reduce the amount of overnight heating required. As such, solar air heaters could be used with the PCM for peak load shifting. However, it is also clear that the indoor temperature dropped below 17 °C overnight, and, as such, some form of supplementary heating will be required.

The benefit of using the solar air heater with PCM is highlighted on days 1 and 2, as shown in Figure 5. Starting from midnight, it is evident that the PCM had stored energy the previous day and was discharging the stored energy overnight. By 1:40 a.m., Hut-1 had dropped below 17 °C. At this point, supplementary heating would be required to maintain comfort. Meanwhile, the lowest temperature in Hut-2 before the hut started heating up again was 18 °C, effectively eliminating the need for overnight heating due to the PCM. During the daylight hours of day 1, there was not enough heat to elevate the temperature in the huts significantly over 21 °C. As a result, the temperature profiles of both huts were similar between 10:50 a.m. and 4:40 p.m. Although a large difference in peak temperatures was not observed, the room temperature of Hut-2 did marginally exceed 20 °C, the maximum melting point of the PCM, and would therefore have been partially charged. This is confirmed by the relatively warmer overnight and early morning temperatures seen in Hut-2 that touched the line of 17 °C at 8:00 a.m. almost 8 h after Hut-1 did. On day 2, the temperature in Hut-2 was always higher than that in Hut-1, despite the solar air heaters providing little to no heat to each hut that day. Instead, the difference in temperatures was due to the PCM continuing to discharge energy from the day before.

### 3.3. Performance of PCM with a Combination of Heat Supplies

Based on the results of the previous sections, it was hypothesised that a combination of passive and active solar heating mechanisms would eliminate the cold nights experienced when using just the solar heater and resolve the issue of extra energy being consumed by Hut-2 when using the oil heater only. The same combination was tested with three different temperature constraints. Results were then judged based on the system’s ability to maintain thermal comfort, and effectively reduce energy consumption.

#### 3.3.1. The First Run of 20–22 °C Temperature Constraints

**Figure 6:**
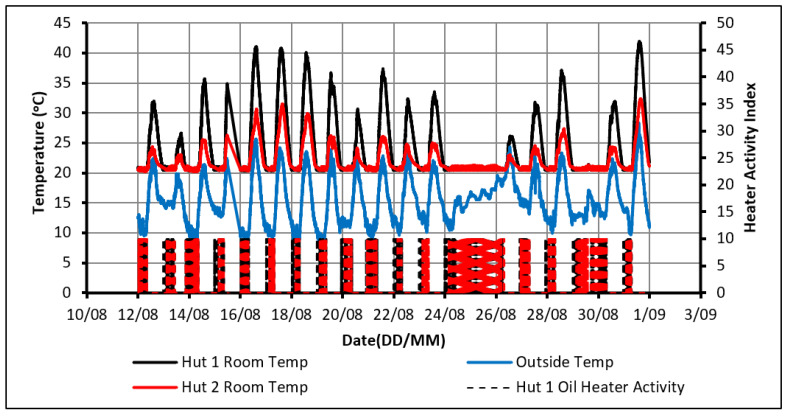
Temperature profiles and oil heater activity (20–22 °C temperature constraints).

Figure 6 shows the interior temperatures within the huts, along with the outdoor temperature and electric heater activity over 20 days. The impact of the electric heater operating under temperature constraints is visible, turning on to prevent the temperature from dropping below 20 °C while not heating them above 22 °C. The electric oil heater was primarily used at night-time except on a few days (13, 14 and 18) when the solar heaters could not provide sufficient warmth. In this respect, the proposed configuration successfully met part of the heating and comfort requirements by preventing temperatures to drop below the minimum comfort level of 17 °C. Although the peak temperature in Hut-2 was noticeably lower, the temperatures within both huts increased rapidly due to a combination of solar air heaters and sunshine irradiance during the daytime.

**Figure 7:**
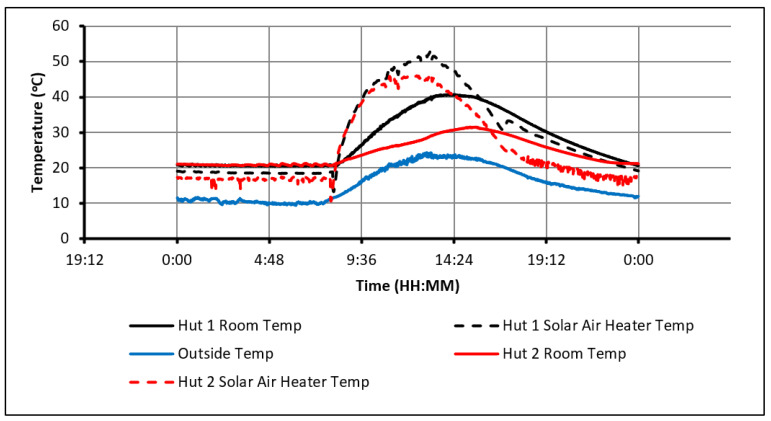
Temperature profiles of the huts and solar air heater (20–22 °C temp constraints, day 6).

Figure 7 shows the 24 h of day 6 and provides an insight into the behaviour of the huts when they overheat. The huts’ temperatures were initially kept between 20 °C and 22 °C overnight by the electric heater. By 8:20 a.m., a rise in temperature due to solar irradiation and the solar air heaters was observed. The temperature in Hut-1 increased at a rapid rate and hit a maximum temperature of 41 °C at 2:40 p.m. The heating in Hut-2 was notably slower due to the charging of the PCM. Despite this, Hut-2 still suffered from overheating and at 2:55 p.m. reached its peak temperature of 30 °C. This occurred because the PCM could not absorb all heat to prevent overheating in Hut-2. As mentioned, the electric heater maintained the room between 20 °C and 22 °C overnight and subsequently prevented the complete discharge of the energy that was stored during the day. Thus, as soon as solar radiation started to heat the room temperatures, the PCM was already partially charged, decreasing the amount of this additional heat that could be absorbed. To rectify this issue, the PCM must be allowed to be fully discharged overnight. One of the recommended actions to tackle this issue is to optimize the quantity/quality of PCM so that larger heat quantity is absorbed during the day, especially when the solar heating is employed.

**Figure 8:**
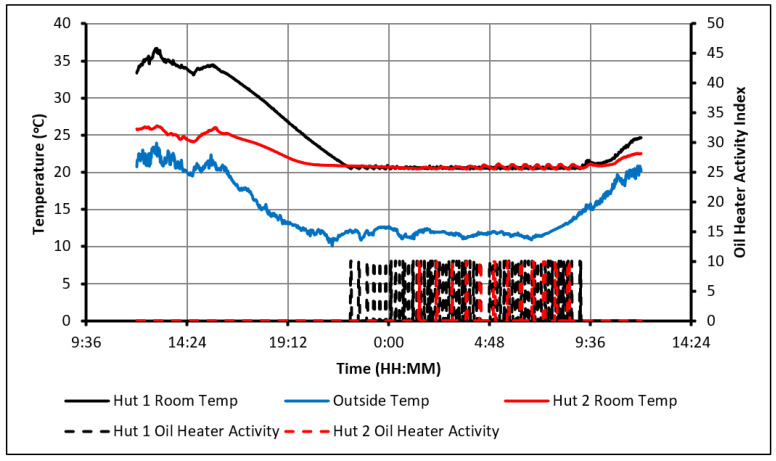
Analysis of heater activity (20–22 °C temperature constraint, day 8 and 9).

As seen in the previous figures, the heaters’ activity shows that the presence of the PCM delayed the cooling of Hut-2 to an extent that it hit the minimum temperature constraint significantly later than Hut-1. As a result, over the 20 full days, an overall energy saving of 33% was achieved in Hut-2. To illustrate how the energy is being saved, the 24 h starting from midday on day 8 are examined in Figure 8.

Both huts were being heated during the day without the use of the electric heater until 3:40 p.m., at which point the indoor temperatures began to drop. During that time, the PCM was being charged and began to discharge, providing heat to Hut-2 and slowing down the rate of temperature drop compared to that observed in Hut-1. As a result, the oil heater in Hut-1 first turned on at 10:10 p.m., while the heater in Hut-2 was only required only after 1:20 a.m., the next morning and this subsequently caused an energy saving of 56%.

**Figure 9:**
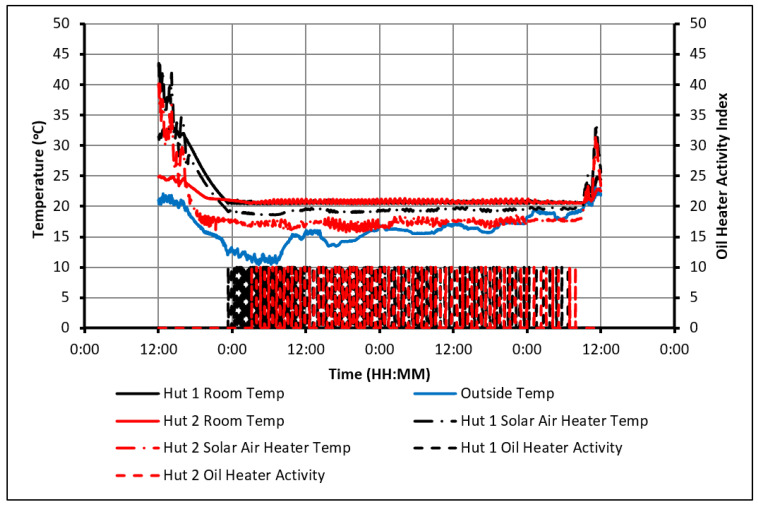
Temperature profiles and heater activity (20–22 °C temp constraints, days 12–15).

Of further interest is the analysis of 72 h shown in Figure 9. The PCM is charged during the first day and the release of this heat that night meant that, while Hut-1 required the electric heater to start at 11:20 p.m. that night, Hut-2 did not need any additional heating until 3:10 a.m. the next morning. However, for the next 54 h, the electric heaters were used in both huts to keep them between 20 °C and 22 °C. As a result, the PCM never had a sufficient chance to release its stored energy and delay the need for the electric heater in Hut-2 as it had done previously. The impact this had on the energy consumption in Hut-2 is obvious when splitting the 72 h into two 36-h periods. In the first half of the period, where the discharging of the PCM was observed, Hut-2 made an energy saving of 36%. However, over the second 36 h, Hut-2 consumed 16.8% more energy than Hut-1. Although an energy saving of 17.1% was observed over the total 72-h period, the second half shows that the configuration has a potential for improvement and can result in excess energy consumption in Hut-2 if there are consecutive cold and cloudy days.

#### 3.3.2. The Second Run of 19–21 °C Temperature Constraints

**Figure 10:**
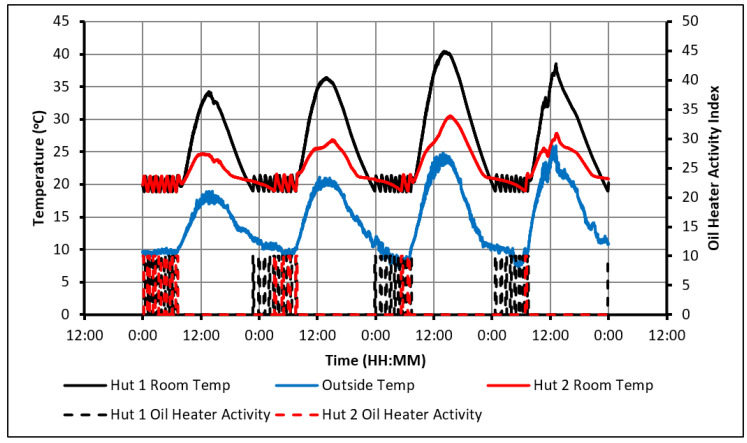
Temperature profiles and heater activity (19–21 °C temperature constraints).

To ensure that the PCM was given a chance to discharge overnight, the temperature constraint of the oil heater was lowered slightly to maintain the indoor temperature between 19 and 21 °C. This range was chosen as it would allow the room to drop below the maximum melting temperature of the PCM, facilitating the discharge of the PCM before partially charging it overnight. The partial charging of the PCM overnight was desired in case the following day was cloudy and the solar air heaters could not fully charge the PCM on their own. The resulting temperature of the huts and outdoor temperature when using this configuration can be seen in Figure 10. Hut-2 took a significantly longer time to drop to 19 °C than Hut-1 each night, which shows that the PCM is discharging while the electric heater was still required. However, it was observed that Hut-2 temperature still exceeded 25 °C on days 2, 3, and 4 when outdoor temperatures exceeded 20 °C.

As calculated, Hut-2 consumed 41.1% less energy than Hut-1 over the 96 h. On nights 2, 3, and 4, there was a significant delay when the huts started using the electric heater. Once again, this was due to the PCM being charged by the solar air heater during the day and releasing the stored energy overnight. On the final night, the oil heater in Hut-1 first turned on at 11:50 p.m., while Hut-2 did not require the heater at all. The fact that the night temperatures were substantially below 15 °C and even below 10 °C on four nights suggests that the discharging of the PCM was enabled by the reduced temperature constraints

#### 3.3.3. The Third Run of 17–19 °C Temperature Constraints

**Figure 11:**
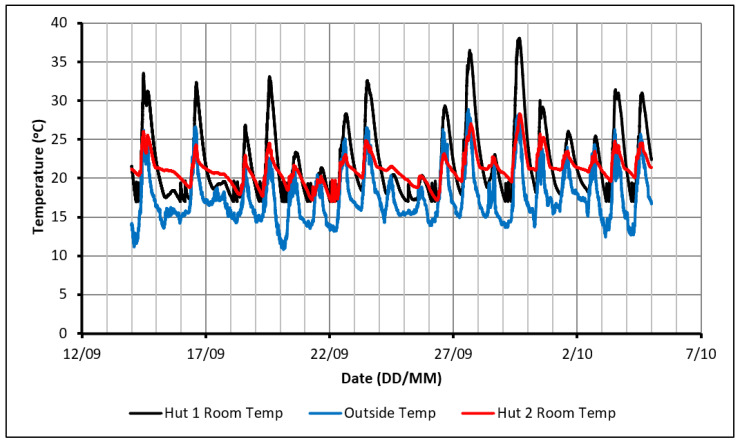
Indoor and outdoor temperature profiles (17–19 °C temperature constraints).

In the third attempt to solve the overheating issue, the temperature range for the oil heater was set to 17–19 °C, which is lower than the maximum melting temperature of PT20. Although 17 °C is right on the verge of the uncomfortable threshold, previous results have indicated that these levels will only be reached in Hut-2 late at night. In the context of a residential building, the residents will likely be asleep at this time and therefore the lower temperatures will have a minimal impact on comfort. Looking at Figure 11, it is observed that both huts are successfully kept at or above 17 °C. Overheating remains a major problem for Hut-1 and was experienced on 14 of the 21 days with a maximum temperature of 38 °C being achieved on day 16. In Hut-2, the PCM demonstrates the benefits of its storage capacity by significantly reducing the peak temperatures. However, Hut-2 still experienced some overheating, with the 25 °C limits being exceeded on 4 days. On closer inspection, on all these days, the temperature in Hut-2 did not drop below 19.5 °C the night prior. As a result, the PCM could not fully discharge and was, therefore, more susceptible to overheating.

**Figure 12:**
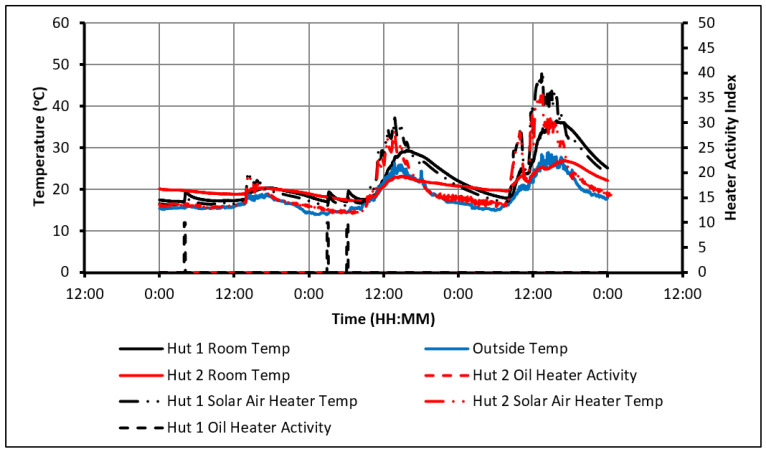
Temperature profiles and heater activity (17–19 °C temp. constraints, days 12–14).

This is examined further in Figure 12, which examines days 12, 13, and 14. On day 12, both huts were only heated to around 20 °C, which is enough to partially charge the PCM. This is made evident by the fact that Hut-2 did not require the oil heater overnight, while Hut-1 did. It is also observed that the room temperature dropped to 17.2 °C overnight, low enough for the PCM to significantly, if not fully, discharge the energy stored on day 12. As a result, when heating began on day 13, the PCM in Hut-2 could store enough energy to prevent overheating. This temperature drop was observed despite the outdoor temperature exceeding 25 °C. However, the following night the room temperature in Hut-2 did not drop below 19.6 °C. This was due to a combination of the warm outdoor temperatures and the significant amount of energy that had been stored in the PCM during day 13, resulting in an incomplete discharge. Subsequently, the still partially discharged PCM could not store enough energy on day 14 to prevent overheating. This highlights that, despite lowered temperature constraints, overheating can occur when there are consecutive hot days and warm nights. The authors also noted that the high temperatures and sunny weather conditions during the testing of this final configuration may not have been fully representative of typical winter conditions, making overheating appear to be a significant issue. If colder nights were experienced, the PCM would have discharged more, preventing overheating. Regardless, the intention is to produce a system that can maintain comfortable temperatures using solar energy and PCM. Although focusing on winter, the solution should be robust enough to deal with unexpected conditions. There are two possible solutions: either it must be ensured that the PCM is fully discharged overnight, or the amount of heating provided to the hut during the day must be controlled. As the study is focused on the winter months, forcing the overnight discharge of the PCM is inadvisable in case the following day is cold or cloudy, and the stored energy is required. Therefore, the most feasible solution is to enforce a temperature ensuring they turn off once the temperature rises between 22 °C and 23 °C.

Over the 21-day period during which this configuration was run, Hut-2 achieved an energy saving of 87.5%. Despite the positive result, the mean outdoor temperature recorded was 17.3 °C, which is higher than the minimum temperature constraint, meaning that the heater would barely be required. This is evident as Hut-1 also only required the heater on 12 nights. In addition, a few nights dropped significantly below 15 °C, meaning that a full large discharge of the PCM may not have been the result of the lower temperature constraints, but rather due to the extra discharge time afforded by a lower rate of cooling. All of this indicates that looking at the entire range of these results does not provide a true representation of the behaviour of the set-up in winter. Instead, examining select days with more appropriate conditions could provide a better insight into the effectiveness of this proposed heating solution.

**Figure 13:**
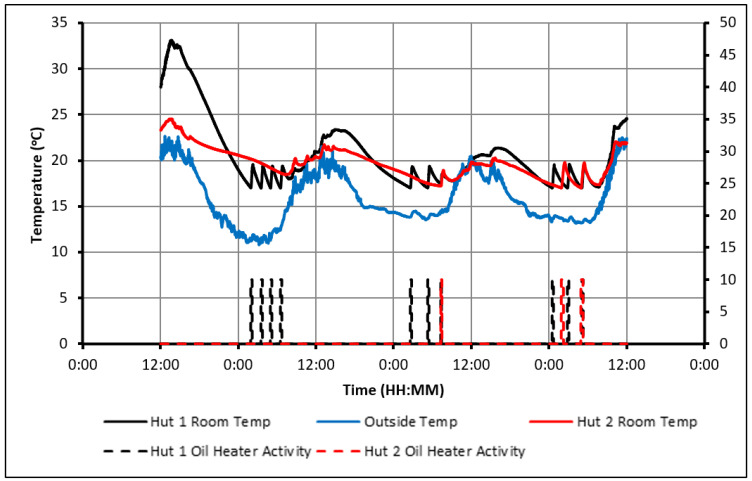
Temperature profiles and heater activity (17–19 °C temp constraints, 72-h period).

Figure 13 cuts down the collected data to only show the results from mid-day on day 6 to mid-day on day 9. Over this period, the average temperature was 16 °C. Although only 1.3 °C is less than the overall average, this is below the temperature constraint and it can be seen that Hut-1 requires the heater each night. This is important because, if Hut-1 does not require any heating, the PCM provides no energy-saving benefits. Looking at the start of Figure 13, it is observed that the PCM was being charged during the day. Once the temperatures dropped, the release of stored energy prevented Hut-2 from needing to use the oil heater. Conversely, the heater in Hut-1 turned on at 1:55 a.m. The minimum temperature reached by Hut-2 that night was 18.5 °C.

Therefore, it can be determined that additional energy was saved in Hut-2 because of lowered temperature constraints. The second day exhibited a similar trend, with the charging of the PCM once again evident, although not to the same extent. Despite a warmer temperature than the previous night, the reduction in stored charge led to the heater being used in Hut-2 at 7:20 a.m. the next morning. However, this was nearly 5 h after Hut-1 and resulted in an energy saving of 69.4%. If a higher temperature constraint had been implemented, more heating would have been required. Over the third day, the room temperature in Hut 2 was even lower than the previous two, barely breaking 20 °C. The difference in peak temperatures shows that some charging did occur, but this was not enough to prevent the heater from being required in Hut-2 that night. On the third night, there was no difference in energy consumption between huts, but the initial use of the heater in Hut-2 was delayed by one and a half hours, which is still useful for peak load shifting. For the three days, the overall energy saving achieved by Hut-2 was 60%. These results clearly show that the use of lower temperature constraints has the potential to significantly improve both peak load shifting and energy consumption in Hut-2 when using a solar air heater and oil heater together.

## 4. Conclusions

This study investigated a solution to successfully maintain thermal comfort and reduce energy consumption within the buildings of lightweight materials using PCM and a combination of heat sources. The combination consisted of passive and active solar heating mechanisms along with a temperature-controlled electric heater. A set of experiments was conducted in two identical huts at the University of Auckland. The only difference between them was that the control Hut-1 was lined with standard gypsum boards while the test Hut-2 was lined with the PCM (PT20) impregnated gypsum boards. The initial experiments of this study investigated the use of individual heat supplies in the presence of the PCM, such as passive solar heating only, and as expected, it was not sufficient to maintain thermal comfort. To make the most out of the presence of PCM, an electric heater was used mostly at night within pre-defined temperature constraints to maintain the comfortable conditions but with an issue of extra energy consumption in Hut-2. When active solar air heating was applied individually, it was not possible to maintain comfortable conditions due to reliability issues that persisted even with the PCM, severely limiting their energy-saving potential. Therefore, the initial experiments proved that the potential use of an individual heat supply was limited, and hence, the solution of using combined heat supplies was investigated to demonstrate significant benefits over a range of weather conditions. The successful application of the combined configuration returned energy savings of around 60% on average and maintained thermal comfort of less fluctuation in indoor temperature. Choosing appropriate temperature constraints (17–19 °C), that allow the complete discharge of the PCM overnight, was the most important experimental factor in the studied set-up. While the authors of this study focused on the temperature constraints method, thermo-physical properties of PCM, as well as thickness of PCM-boards and the PCM quantity, could be optimised for better results.

## Data Availability

Not applicable.
